# Shape analysis of the amygdala, hippocampus and thalamus in former American football players

**DOI:** 10.1093/braincomms/fcaf440

**Published:** 2025-11-07

**Authors:** Omar John, Alana Wickham, Leonard B Jung, Anya S Mirmajlesi, Jared Stearns, Katherine Breedlove, Nicholas Kim, Daniel H Daneshvar, Tashrif Billah, Ofer Pasternak, Arushi Chamaria, Michael J Coleman, Yorghos Tripodis, Charles H Adler, Charles Bernick, Laura J Balcer, Richard Jarrett Rushmore, Michael L Alosco, Inga K Koerte, Alexander P Lin, Jeffrey L Cummings, Eric M Reiman, Robert A Stern, Martha E Shenton, Hector Arciniega, Sylvain Bouix, for the DIAGNOSE CTE Research Project, Eric Reiman, Yi Su, Kewei Chen, Hillary Protas, Connie Boker, Michael L Alosco, Rhoda Au, Robert C Cantu, Lindsay Farrer, Robert Helm, Douglas I Katz, Neil Kowall, Jesse Mez, Gustavo Mercier, James Otis, Robert A Stern, Jason Weller, Irene Simkin, Alondra Andino, Shannon Conneely, Courtney Diamond, Tessa Fagle, Olivia Haller, Tennyson Hunt, Nicole Gullotti, Megan Mariani, Brian Mayville, Kathleen McLaughlin, Mary Nanna, Taylor Platt, Surya Pulukuri, Fiona Rice, Madison Sestak, Michael McClean, Yorghos Tripodis, Douglas Annis, Christine Chaisson, Diane B Dixon, Carolyn Finney, Kerrin Gallagher, Kaitlin Hartlage, Jun Lu, Brett Martin, Emmanuel Ojo, Joseph N Palmisano, Brittany Pine, Janani Ramachandran, Sylvain Bouix, Jennifer Fitzsimmons, Alexander P Lin, Inga K Koerte, Ofer Pasternak, Martha E Shenton, Hector Arcinieago, Tashrif Billah, Elena Bonke, Katherine Breedlove, Eduardo Coello, Michael J Coleman, Leonhard Jung, Huijun Liao, Maria Loy, Elizabeth Rizzoni, Vivian Schultz, Annelise Silva, Brynn Vessey, Tim L T Wiegand, Sarah Banks, Charles Bernick, Jason Miller, Aaron Ritter, Marwan Sabbagh, Raelynn de la Cruz, Jan Durant, Morgan Golceker, Nicolette Harmon, Kaeson Kaylegian, Rachelle Long, Christin Nance, Priscilla Sandoval, Robert W Turner, Kenneth L Marek, Andrew Serrano, Charles H Adler, David W Dodick, Yonas Geda, Jennifer V Wethe, Bryce Falk, Amy Duffy, Marci Howard, Michelle Montague, Thomas Osgood, Debra Babcock, Patrick Bellgowan, Laura Balcer, William Barr, Judith Goldberg, Thomas Wisniewski, Ivan Kirov, Yvonne Lui, Charles Marmar, Lisena Hasanaj, Liliana Serrano, Alhassan Al-Kharafi, Allan George, Sammie Martin, Edward Riley, William Runge, Jeffrey L Cummings, Elaine R Peskind, Elizabeth Colasurdo, Daniel S Marcus, Jenny Gurney, Richard Greenwald, Keith A Johnson

**Affiliations:** Department of Rehabilitation Medicine, NYU Grossman School of Medicine, New York, NY 10016, USA; NYU Langone Concussion Center, NYU Langone Health, New York, NY 10016, USA; Psychiatry Neuroimaging Laboratory, Brigham and Women’s Hospital, Harvard Medical School, Boston, MA 02145, USA; Psychiatry Neuroimaging Laboratory, Brigham and Women’s Hospital, Harvard Medical School, Boston, MA 02145, USA; Psychiatry Neuroimaging Laboratory, Brigham and Women’s Hospital, Harvard Medical School, Boston, MA 02145, USA; cBRAIN, Department of Child and Adolescent Psychiatry, Psychosomatics, and Psychotherapy, University Hospital, Ludwig-Maximilians-Universität, Munich, 80336, Germany; Department of Neurosurgery, University Hospital, Ludwig-Maximilians-Universität, Munich, 80336,Germany; Department of Rehabilitation Medicine, NYU Grossman School of Medicine, New York, NY 10016, USA; NYU Langone Concussion Center, NYU Langone Health, New York, NY 10016, USA; Department of Rehabilitation Medicine, NYU Grossman School of Medicine, New York, NY 10016, USA; NYU Langone Concussion Center, NYU Langone Health, New York, NY 10016, USA; Center for Clinical Spectroscopy, Department of Radiology, Brigham and Women’s Hospital, Harvard Medical School, Boston, MA 02115, USA; Psychiatry Neuroimaging Laboratory, Brigham and Women’s Hospital, Harvard Medical School, Boston, MA 02145, USA; Department of Physical Medicine and Rehabilitation, Harvard Medical School, Boston, MA 02115, USA; Department of Physical Medicine and Rehabilitation, Massachusetts General Hospital, Boston, MA 02114, USA; Department of Physical Medicine and Rehabilitation, Spaulding Rehabilitation Hospital, Boston, MA 02129, USA; Psychiatry Neuroimaging Laboratory, Brigham and Women’s Hospital, Harvard Medical School, Boston, MA 02145, USA; Psychiatry Neuroimaging Laboratory, Brigham and Women’s Hospital, Harvard Medical School, Boston, MA 02145, USA; Department of Radiology, Brigham and Women’s Hospital, Harvard Medical School, Boston, MA 02115, USA; Department of Psychiatry, Massachusetts General Hospital, Boston, MA 02114, USA; Psychiatry Neuroimaging Laboratory, Brigham and Women’s Hospital, Harvard Medical School, Boston, MA 02145, USA; Psychiatry Neuroimaging Laboratory, Brigham and Women’s Hospital, Harvard Medical School, Boston, MA 02145, USA; Department of Biostatistics, Boston University School of Public Health, Boston, MA 02118, USA; Department of Neurology, Mayo Clinic College of Medicine, Mayo Clinic Arizona, Scottsdale, AZ 85259, USA; Cleveland Clinic Lou Ruvo Center for Brain Health, Las Vegas, NV 89106, USA; Department of Neurology, University of Washington, Seattle, WA 98195, USA; Department of Neurology, NYU Grossman School of Medicine, New York, NY 10016, USA; Department of Population Health, NYU Grossman School of Medicine, New York, NY 10017, USA; Department of Ophthalmology, NYU Grossman School of Medicine, New York, NY 10017, USA; Psychiatry Neuroimaging Laboratory, Brigham and Women’s Hospital, Harvard Medical School, Boston, MA 02145, USA; Department of Anatomy and Neurobiology, Boston University Chobanian & Avedisian School of Medicine, Boston, MA 02118, USA; Department of Neurology, Boston University Alzheimer’s Disease Research Center and CTE Center, Boston University Chobanian & Avedisian School of Medicine, Boston, MA 02118, USA; Psychiatry Neuroimaging Laboratory, Brigham and Women’s Hospital, Harvard Medical School, Boston, MA 02145, USA; cBRAIN, Department of Child and Adolescent Psychiatry, Psychosomatics, and Psychotherapy, University Hospital, Ludwig-Maximilians-Universität, Munich, 80336, Germany; Graduate School of Systemic Neurosciences, Ludwig-Maximilians-Universität, Munich, Bavaria, 82152, Germany; Center for Clinical Spectroscopy, Department of Radiology, Brigham and Women’s Hospital, Harvard Medical School, Boston, MA 02115, USA; Department of Radiology, Brigham and Women’s Hospital, Harvard Medical School, Boston, MA 02115, USA; Chambers-Grundy Center for Transformative Neuroscience, Pam Quirk Brain Health and Biomarker Laboratory, Department of Brain Health, Kirk Kerkorian School of Medicine, University of Nevada Las Vegas, Las Vegas, NV 89154, USA; Banner Alzheimer’s Institute and Arizona Alzheimer’s Consortium, Phoenix, AZ 85006, USA; Department of Psychiatry, University of Arizona, Phoenix, AZ 85004, USA; Department of Psychiatry, Arizona State University, Phoenix, AZ 85008, USA; Neurogenomics Division, Translational Genomics Research Institute and Alzheimer’s Consortium, Phoenix, AZ 85004, USA; Department of Anatomy and Neurobiology, Boston University Chobanian & Avedisian School of Medicine, Boston, MA 02118, USA; Department of Neurology, Boston University Alzheimer’s Disease Research Center and CTE Center, Boston University Chobanian & Avedisian School of Medicine, Boston, MA 02118, USA; Department of Neurosurgery, Boston University Chobanian & Avedisian School of Medicine, Boston, MA 02118, USA; Psychiatry Neuroimaging Laboratory, Brigham and Women’s Hospital, Harvard Medical School, Boston, MA 02145, USA; Department of Radiology, Brigham and Women’s Hospital, Harvard Medical School, Boston, MA 02115, USA; Department of Psychiatry, Massachusetts General Hospital, Boston, MA 02114, USA; Department of Rehabilitation Medicine, NYU Grossman School of Medicine, New York, NY 10016, USA; NYU Langone Concussion Center, NYU Langone Health, New York, NY 10016, USA; Institute for Translational Neuroscience, NYU Grossman School of Medicine, New York, NY 10016, USA; Department of Software Engineering and Information Technology, École de Technologie Supérieure, Université du Québec, Montréal, QC, H3C 1K3, Canada

**Keywords:** neuroimaging, structural MRI, shape analysis, sports-related head injury, repetitive head impacts

## Abstract

Repetitive head impacts are common in contact and collision sports and are linked to structural brain changes and an elevated risk of neurodegenerative diseases such as Chronic Traumatic Encephalopathy. Identifying early in vivo structural markers remains challenging. Although diagnosis currently requires post-mortem confirmation, clinical symptoms, including cognitive impairment and behavioural changes, are reflected in the diagnosis of Traumatic Encephalopathy Syndrome. These symptoms align with dysfunction in key brain regions—amygdala, hippocampus and thalamus—which support memory, emotion and behaviour and commonly show tau pathology in Chronic Traumatic Encephalopathy. This study uses shape analysis to examine structural differences in these regions between former American football players and unexposed asymptomatic controls and evaluates the influence of age, head impact exposure and clinical diagnosis on brain structure. We analyzed brain morphology in former American football players (*n* = 163) and unexposed, asymptomatic controls (*n* = 53). Structural segmentation was performed with FreeSurfer 7.1, and the shape analysis pipeline was used to generate subregional reconstructions. Vertex-level morphometry, based on the logarithm of the Jacobian determinant and radial distance, quantified local surface area dilation and thickness. Group differences were examined with covariate-adjusted linear regression models contrasting football players and controls, as well as participants with and without a Traumatic Encephalopathy Syndrome diagnosis. Partial correlations examined the influence of age, age of first football exposure and cumulative head impact index metrics, including frequency, linear acceleration and rotational force. Models were adjusted accordingly for age, body mass index, education, race, imaging site, apolipoprotein ϵ4 status and total intracranial volume. Former football players exhibited bilateral surface area contractions in the hippocampus and amygdala, along with reduced amygdala thickness, compared to controls. Older age was associated with widespread surface contractions and thinning across all regions, except for preserved thickness in the left hippocampus. An earlier age of first exposure to football correlated with surface contractions in the thalamus and left hippocampus. Greater cumulative linear acceleration was linked to bilateral hippocampal surface contractions and reduced thickness in the left thalamus, while greater rotational force exposure was associated with hippocampal thinning. No significant structural differences were found between players with and without a diagnosis of Traumatic Encephalopathy Syndrome. These findings extend volume-based research by revealing localized alterations in surface area dilation and thickness and emphasize the roles of age and repetitive head impact exposure in long-term brain changes.

## Teaser

This study demonstrates that former American football players showed localized shape alterations in the hippocampal, amygdala and selective thalamic changes relative to controls. Earlier first exposure to football and higher cumulative head-impact accelerations were associated with greater changes. Findings suggest that surface-based shape metrics may provide sensitive *in vivo* markers of exposure to repetitive head impacts.

## Graphical Abstract

**Graphical Abstract:**
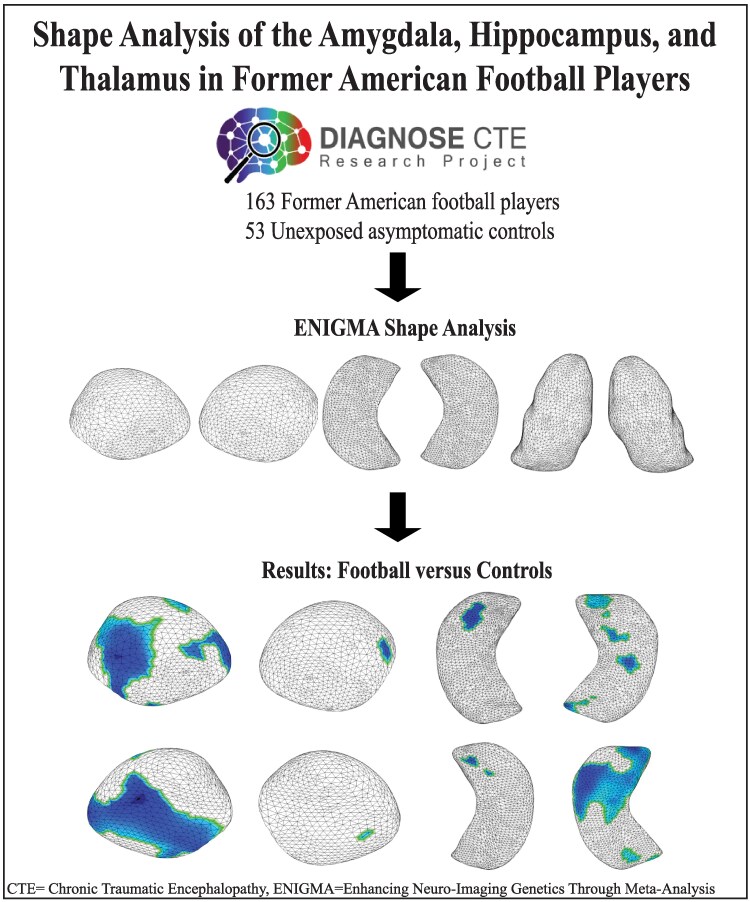

## Introduction

Athletes in contact and collision sports, such as American football, frequently experience repetitive head impacts (RHI), which have been linked to alterations in brain function, disrupted neural connectivity, cognitive deficits and an increased risk of neurodegenerative tauopathies, including Chronic Traumatic Encephalopathy (CTE)^1-15^ Multiple RHI-related factors, including a younger age of first exposure to tackle football, total years of play and cumulative head impact burden, have been linked to worse long-term neurological outcomes and an increased likelihood of a post-mortem CTE diagnosis.^8,16-18^ Among these, total years of football participation is frequently used as a proxy for RHI exposure and has demonstrated a dose–response relationship with both the probability and severity of CTE pathology.^18^ Furthermore, in former American football players, career-long estimates of head impacts have been directly correlated with greater CTE-related neurofibrillary tangle burden, reinforcing the connection between cumulative RHI exposure and underlying neuropathological changes.^17^

The neuropathology of CTE is well-characterized post-mortem and is defined by hyperphosphorylated tau (*P*-tau) accumulation in perivascular spaces and regional brain atrophy.^7,9,11,12,14,16,19-22^ The disease follows a staged progression (Stages I–IV), with increasing severity of neuropathology.^23,24^ Despite extensive post-mortem documentation,^7,9,11,14-16,19-22,25-27^ there remains a critical lack of *in vivo* biomarkers for diagnosing CTE, which is essential for developing interventions to slow or prevent disease progression. Current efforts to establish an *in vivo* diagnostic measure include evaluating clinical features associated with neuropathologically diagnosed CTE, as outlined in the 2021 National Institute of Neurological Disorders and Stroke (NINDS) consensus diagnostic criteria for Traumatic Encephalopathy Syndrome (TES).^28^ However, it is important to note that TES diagnosis relies solely on clinical symptomatology and does not incorporate fluid or neuroimaging biomarkers for additional confirmation. Therefore, focusing on neuroanatomical features consistently observed at post-mortem in CTE is crucial for advancing *in vivo* biomarkers and refining the clinical diagnosis of TES.

One way to study potential CTE in living individuals is by assessing high-risk populations, such as former American football players, and examining morphometric changes in key brain regions associated with the disease. Particularly, structural magnetic resonance imaging (MRI) may be a potential tool for identifying neuroanatomical markers of CTE. We previously identified significant volumetric reductions in brain regions associated with CTE neuropathology in living former American football players when compared to controls with no history of RHI exposure.^29^ While these findings highlight significant volumetric differences in key regions typically affected by CTE, including the amygdala, hippocampus, entorhinal cortex, insula sulcus, temporal pole and superior frontal gyrus, they do not capture more nuanced and detailed subregional differences that may provide a more direct link to well-documented post-mortem findings of CTE.^14,19,20^ The Enhancing Neuro-Imaging Genetics Through Meta-Analysis (ENIGMA) Shape Analysis pipeline captures morphometric changes in brain structures at the subregional level. This approach has proven effective in major depressive disorder,^30^ substance dependence,^31^ schizophrenia,^32^ 22q11.2 deletion syndrome,^33^ and mild traumatic brain injury (TBI).^34^ The ENIGMA shape-analysis framework is well-suited to detect fine-grained, region-specific alterations in structures implicated in CTE, including the amygdala, hippocampus and thalamus. These regions are repeatedly implicated in the disease, with tau accumulation, focal atrophy and associated neuropsychiatric symptoms, making a surface-based approach advantageous for capturing subtle patterns that volumetric measures may miss. Although these regions are not unique to CTE, their established histopathological involvement and functional relevance (e.g. memory, mood regulation, arousal and stress reactivity) make them particularly informative for testing whether *in vivo* neuroimaging can detect changes that align with the disease process. Thus, while volumetry captures overall size differences in structures, shape analysis can provide spatially localized information on subregional abnormalities, offering novel insight into areas disproportionately affected by RHI and aligning with the neuropathology of CTE. By anchoring our analyses to regions with strong post-mortem evidence, our study directly tests whether neuroimaging can detect the most biologically relevant signatures of CTE in living individuals, thereby providing a focused and translationally meaningful starting point for potential biomarker development.

In this study, we aim to investigate the morphometric properties of three brain regions—amygdala, hippocampus and thalamus—that are known to be impacted by RHI and are associated with CTE neuropathology. We hypothesize that former American football players will show significant morphological changes in these regions compared to unexposed asymptomatic controls, indicating potential structural alterations linked to RHI and the possible development of CTE. Additionally, we aim to examine how age and factors related to RHI exposure, such as the age of first exposure to tackle football and career-long estimates of head impacts, contribute to subregion-level structural changes. We hypothesize that greater age and earlier exposure to tackle football and higher estimates of cumulative head impacts will correlate with more pronounced morphometric changes in the amygdala, hippocampus and thalamus. Finally, we aimed to assess group-level differences between former American football players with and without a TES diagnosis to further explore the relationship between morphometric alterations and clinical symptoms linked to CTE. We hypothesize that former American football players with a TES diagnosis will exhibit greater structural changes in these regions compared to those without the diagnosis, suggesting a link between morphometric changes and worse clinical symptoms linked with CTE.

## Materials and methods

### Study design and participants

This study is part of the Diagnostics, Imaging and Genetics Network for the Objective Study and Evaluation of Chronic Traumatic Encephalopathy (DIAGNOSE CTE) Research Project.^35^ A total of 240 male participants were enrolled in the study, including 180 former American football players (120 former professional football players and 60 former college players) and 60 asymptomatic control participants who had no history of RHI exposure. Detailed methods for inclusion and exclusion criteria are published elsewhere.^35^

Baseline data collection occurred from September 2016 to February 2020 at one of four US sites: Boston University Chobanian & Avedisian School of Medicine (MRI data collection at Brigham and Women’s Hospital), New York University Langone Medical Center, Cleveland Clinic Lou Ruvo Center for Brain Health in Las Vegas and Mayo Clinic Arizona positron emission tomography (PET) data collection at Banner Alzheimer’s Institute. All participants took part in an evaluation spanning three days, consisting of clinical history, neurological and neuropsychological examinations, MRI and PET imaging, lumbar puncture and venipuncture, as well as participant- and informant-based functional dependence, mood and behaviour questionnaires. All study sites received approval from their Institutional Review Board, and all study participants and their partners provided written informed consent before enrollment.

Data from 22 participants (17 former American football players and 5 unexposed asymptomatic controls) were excluded from the analyses because of poor-quality structural MRI (*n* = 12), missing scans (*n* = 9), or a duplicate scan error in 1 participant. Two additional unexposed asymptomatic controls were excluded from the analysis after discovering they did meet the exclusion criteria (pre-existing psychiatric conditions or participation in high school football). The final sample of former American football players consisted of 163 players (111 former professional and 52 former college players) and 53 unexposed asymptomatic controls.

### Sample characteristics

Semi-structured interviews and online questionnaires gathered information on demographics (e.g. age, education, racial identity and ethnicity), clinical history, athletic history, military involvement and brain injury history (see Table 1 for all cohort characteristics used in this study).

**Table 1:** Cohort characteristics

	Former football players (*n* = 163)	Unexposed asymptomatic Controls (*n* = 53)
Age	57.3 y (8.2), [45–74 y]	59.5 y (8.3), [45–74 y]
BMI kg/m^2^	32.6 (4.8), [22.8–47.4]	30.9 (4.7), [23.7–43.5]
Education	16.8 y (1.5), [15–27 y]	17.3 y (3.4), [13–30 y]
Apolipoprotein ϵ4 carriers^a^	44 (27%)	10 (18.9%)
Race		
White	104 (63.8%)	34 (64.2%)
Black/African American	56 (34.4%)	18 (34%)
American Indian/Alaska Native	0 (0%)	0 (0%)
Asian	0 (0%)	0 (0%)
Native Hawaiian/Other Pacific Islander	0 (0%)	1 (1.9%)
Multiple races	3 (1.8%)	0 (0%)
Exposure to RHIs		
Age of first exposure to football^b,c^	11.1 y (2.9), [4 y–18 y]	
Cumulative head impact index seasons		
Frequency^b^	10 907 (4739), [3560–28 020]	
Linear acceleration^b^	228 978 (72 935), [79 213–446 257]	
Rotational force^b^	18 386 688 (6 414 761), [6 053 874–44 072 194]	
Traumatic encephalopathy syndrome diagnosis	105 (64.4%)	

Overview of cohort characteristics, including demographics of 163 former football players and 53 unexposed asymptomatic control participants.

^a^APOE4-carrier analysis was only available for 210 participants.

^b^Values represent [mean, standard deviation (range)].

^c^Years = y.

### Magnetic resonance imaging

#### Image acquisition

All study participants completed a head MRI at one of the four sites or their corresponding imaging centers (see above). All scans followed the same multi-sequence neuroimaging protocol and used the same 3T scanner model (Siemens Magnetom Skyra, Erlangen, Germany; software version VE11) with a 20-channel head coil across the four sites. The protocol included structural, diffusion and functional MRI scans. Relevant to this study are the high resolution (1 × 1 × 1 mm^3^) 3D T1-weighted (T1w) magnetization-prepared-rapid-gradient-echo (MPRAGE) sequence (inversion time = 1100 ms, TR = 2530 ms, TE = 3.36 ms, 7-degree flip angle, 256 FOV) and the high resolution (1 × 1 × 1 mm^3^) 3D T2-weighted (T2w) Sampling-Perfection-with-Application-optimized-Contrasts-by-using-flip-angle-Evolution (SPACE) (TR = 3200 ms, TE = 412 ms, 256 FOV).

#### Image processing

The raw images were visually inspected for complete brain coverage, distortion and motion artifacts using 3D Slicer.^36^ Brain masking was performed on all T1w and T2w scans using custom tools developed by the Psychiatry Neuroimaging Laboratory^37,38^ and further processed with FreeSurfer v7.1^39^ to generate segmentations. Additionally, whole hippocampus and amygdala volumetric measures were calculated separately using a recent hippocampal subfield segmentation method (*recon-all -hippocampal-subfields-T1T2).*^40^

#### Selection of regions of interest

The amygdala, hippocampus and thalamus were selected for analyses *a priori* based on the literature on post-mortem CTE pathology^7,9,12-14,18-20,41,42^ and our previous volumetric findings.^29^ Atrophy in these regions has been observed in post-mortem CTE neuropathology and has been associated with exposure to RHI in *in vivo* neuroimaging studies.^43,44^ Other subcortical regions available for analysis using the ENIGMA Shape Analysis pipeline, including the caudate, putamen, globus pallidus and nucleus accumbens, were not included in the present study. Instead, we prioritized regions with a consistently high neurofibrillary tau burden in post-mortem CTE staging criteria and those with direct clinical relevance to TES. This targeted approach allowed us to focus on structures most strongly linked to CTE neuropathology and clinical symptomatology, rather than broadly examining regions that, while potentially affected, show less disease specificity.

#### Shape analysis

Using the ENIGMA Shape Analysis pipeline, we conducted morphometric analysis on the resulting regions of interest.^45^ This pipeline generates mesh representations of regions of interest using the Medial Demons method, which matches their shape curvatures and medial features to a master template.^46-48^ Next, following the ENIGMA Shape Analysis procedure, expert raters (O.J., A.C. and H.A.) conducted visual quality assurance of the shapes generated for each region and participant. No meshes were discarded due to shape quality. Across the surface of each region of interest, two vertex-level measures of shape morphometry were derived: the logarithm of the Jacobian determinant and radial distance. The logarithm of the Jacobian determinant quantifies local surface dilation, indicating whether a structure’s surface has expanded or contracted at each vertex relative to a template. Negative values represent local surface contraction, whereas positive values represent local expansion. In contrast, radial distance (also termed ‘thickness’) measures the distance from each surface vertex to the structure’s central skeletal axis. Negative values reflect thinning or inward displacement towards the axis, while positive values reflect thickening or outward displacement. Together, these measures provide complementary information: Jacobian determinant reflects local surface area change, whereas radial distance reflects local thickness relative to the structure’s midline.

### Age

We evaluated age within our groups as an independent variable of interest to better understand how structural brain changes vary with aging in both former American football players and unexposed asymptomatic controls. Doing so allows us to assess whether aging trajectories in individuals with RHI exposure deviate from patterns of typical neurodegeneration or normal aging. This may offer additional insight into how the brains of former American football players are aging compared to those of individuals without RHI exposure.

### Exposure to RHI

We evaluated the age at which individuals began participating in organized tackle football to assess the potential effects of early involvement and exposure to RHI.^44,49-52^ Additionally, we used cumulative head impact index (CHII) scores of frequency, linear acceleration and rotational force to investigate their estimated effects on the morphometry of former American football players. CHII scores are estimated by taking into account the total seasons played, player position at each career level and the estimated head impact profiles of these positions based on previous studies that utilized helmet accelerometry data acquired from an open-source tool.^17^ These scores provide an approximation of impact frequency, linear acceleration and rotational force sustained throughout a former player’s American football career. Higher CHII scores reflect increased exposure to RHI.

### TES diagnosis

All study participants were evaluated for a TES diagnosis (yes/no) based on the 2021 NINDS Consensus Diagnosis criteria.^28^ Each participant’s diagnosis was adjudicated through a multidisciplinary diagnostic consensus conference, where panelists reviewed their medical, neurological and psychiatric histories; football exposure and other RHI and TBI history; reports from the participant and informants of functional dependence status and complaints of cognitive, mood and behaviour problems; findings of neurological/motor examinations; and results from standardized neuropsychological tests.

### Statistical analysis

#### Group-level differences

We conducted a linear regression analysis to evaluate group-level differences between former American football players and unexposed asymptomatic control participants. In the model, we controlled for age,^53-55^ body mass index (BMI),^56-58^ race,^55,59^ education years,^60-63^ imaging site, total intracranial volume,^64,65^ and apolipoprotein ϵ4 (APOE4) allele carrier status.^66,67^ We selected these covariates based on their known effects on aging, imaging analysis, or volumetric measures. Throughout our analyses, we present the Jacobian determinant and radial distance results, as well as the average and range of effect sizes (Cohen’s d), along with adjusted *P*-values. The *P*-values are corrected for multiple comparisons using the Benjamini–Hochberg procedure, controlling the false discovery rate (FDR) at q < 0.05. Additionally, we report the significant percentage of the total region, as we did not limit our results to contiguous significant vertices.

#### Age and exposure metrics

We performed partial correlation analyses to assess interactions between our regions of interest (amygdala, hippocampus and thalamus) and age, as well as exposure factors, including age of first exposure to football, and CHII scores of frequency, linear acceleration and rotational force. All exposure measures were evaluated as continuous variables. In the partial correlation analyses, we controlled for the continuous variables of age, body mass index, education years and total intracranial volume. Finally, we evaluated group-level differences between former American football players with and without TES using the linear regression model described above. The effects of age were examined within groups only and we assessed data only from the former American football player group for the analyses conducted on exposure factors and TES. The statistical outputs were visualized by mapping the effect sizes of the significant vertices in each region of interest onto their respective templates. As mentioned above, all *P*-values were corrected for multiple comparisons using the Benjamini–Hochberg FDR procedure, and all adjusted alphas < 0.05 were considered significant. We report Jacobian determinant and radial distance results, along with effect sizes (Cohen’s d) and the percentage of each region showing significance, without applying a spatial extent threshold.

## Results

### Demographic differences

Former American football players had a statistically significantly higher BMI than unexposed asymptomatic controls (Welch two-sample *t*-test (t(89) = 2.3, mean difference = 1.7, 95% CI [0.3, 3.2], *P* = 0.022)). No other variables demonstrated significant differences between the groups; see Table 1 for full demographic details.

### Group-level differences

**Figure 1:**
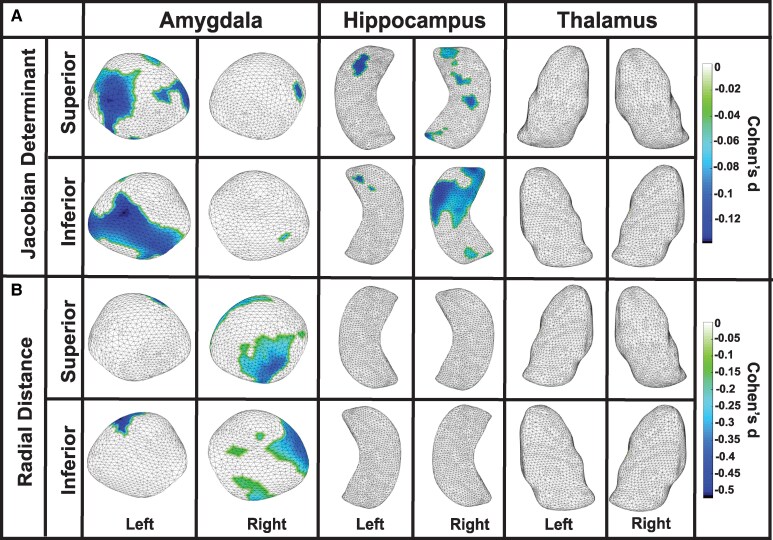
**Structural alterations associated with RHI exposure**. Linear regression analyses demonstrating surface area contractions and thickness reductions in former American football players (*N* = 157) compared to unexposed asymptomatic controls (*N* = 51). (**A**) As assessed by the Jacobian determinant, surface area contractions were revealed via negative values in the amygdala and hippocampus. In the amygdala, left-side contractions were observed in the basolateral, basomedial, centromedial and lateral nuclei (Cohen’s d = −0.101, *P* = 0.02), while right-side contractions were found in the basomedial and lateral nuclei (Cohen’s d = −0.103, *P* < 0.001). In the hippocampus, left-side contractions were noted in the CA1, CA2 and subiculum subregions (Cohen’s d = −0.112, *P* = 0.001), while right-side contractions were observed in the CA1, CA2, CA3/DG and subiculum (Cohen’s d = −0.084, *P* = 0.01). (**B**) Thickness reductions, as assessed by radial distance, showed negative values in the amygdala. Left-side reductions were observed in the basolateral, basomedial and lateral nuclei (Cohen’s d = −0.419, *P* = 0.001), while right-side reductions were found in the basolateral, basomedial, centromedial and lateral nuclei (Cohen’s d = −0.269, *P* = 0.01). DG, dentate gyrus.

In our analysis of the Jacobian determinant, which reflects surface area dilation, former American football players exhibited significant negative values bilaterally in the amygdala and hippocampus, indicating surface area contraction, compared to unexposed asymptomatic controls. In the amygdala, left-side contractions were observed in the basolateral, basomedial, centromedial and lateral nuclei (Cohen’s d = −0.101, *P* = 0.02), while right-side contractions were found in the basomedial and lateral nuclei (Cohen’s d = −0.103, *P* < 0.001). In the hippocampus, left-side contractions were observed in the CA1, CA2 and subiculum subregions (Cohen’s d = −0.112, *P* = 0.001), while right-side contractions were found in the CA1, CA2, CA3/DG and subiculum subregions (Cohen’s d = −0.084, *P* = 0.01). No other regions reached significance; see Fig. 1 and Table 2 for details.

**Table 2:** Group-level differences and interactions

Statistical model	Region of interest		Jacobian determinant (surface dilation ratio)			Radial distance (thickness)		
			Percent of total region (%)	Avg effect size (Cohen’s d)	Effect size range	Percent of total region (%)	Avg effect size (Cohen’s d)	Effect size range
Group Level: Former American Football Players × Unexposed Asymptomatic Controls	Left	Amygdala	34.14	−0.101	−0.057 to −0.138	3.07	−0.419	−0.291 to −0.521
		Hippocampus	2.72	−0.112	−0.091 to −0.132	0	n.s.	n.s.
		Thalamus	0	n.s.	n.s.	0	n.s.	n.s.
	Right	Amygdala	1.32	−0.103	−0.083 to −0.115	28.29	−0.269	−0.116 to −0.429
		Hippocampus	24.62	−0.084	−0.051 to −0.128	0	n.s.	n.s.
		Thalamus	0	n.s.	n.s.	0	n.s.	n.s.
Interaction: Age × Former American Football Players	Left	Amygdala	75.37	−0.007	−0.003 to −0.012	72.81	−0.019	−0.005 to −0.037
		Hippocampus	45.28	−0.008	−0.004 to −0.012	0	n.s.	n.s.
		Thalamus	88.41	−0.004	−0.002 to −0.010	75.30	−0.021	0.009 to −0.044
	Right	Amygdala	78.14	−0.006	−0.003 to −0.011	76.97	−0.016	−0.004 to −0.032
		Hippocampus	74.30	−0.007	0.007 to −0.015	58.15	−0.022	0.014 to −0.047
		Thalamus	79.02	−0.004	0.008 to −0.010	70.70	−0.018	0.009 to −0.044
Interaction: Age of First Exposure	Left	Amygdala	0	n.s.	n.s.	0	n.s.	n.s.
		Hippocampus	0.64	0.026	0.019–0.031	0	n.s.	n.s.
		Thalamus	6.24	0.009	0.006–0.012	0.12	0.040	0.038–0.042
	Right	Amygdala	0	n.s.	n.s.	0	n.s.	n.s.
		Hippocampus	0	n.s.	n.s.	0	n.s.	n.s.
		Thalamus	2.68	0.010	0.008–0.013	0	n.s.	n.s.
Interaction: CHII-Linear Acceleration	Left	Amygdala	0	n.s.	n.s.	0	n.s.	n.s.
		Hippocampus	15.35	−1.22×10^−6^	1.13×10^−6^ to −2.75×10^−6^	56.67	−2.49×10^−6^	6.44×10^−6^ to −1.78×10^−5^
		Thalamus	0	n.s.	n.s.	1.72	−2.18×10^−6^	−1.84×10^−6^ to −2.71×10^−6^
	Right	Amygdala	0	n.s.	n.s.	0	n.s.	n.s.
		Hippocampus	56.31	−5.61×10^−7^	−3.05×10^−7^ to −1.12×10^−6^	23.98	−2.19×10^−6^	1.68×10^−6^ to −3.86×10^−6^
		Thalamus	0	n.s.	n.s.	0	n.s.	n.s.
Interaction: CHII-rotational force	Left	Amygdala	0	n.s.	n.s.	0	n.s.	n.s.
		Hippocampus	0	n.s.	n.s.	11.35	−4.65×10^−8^	5.75×10^−8^ to −1.64×10^−7^
		Thalamus	0	n.s.	n.s.	0	n.s.	n.s.
	Right	Amygdala	0	n.s.	n.s.	0	n.s.	n.s.
		Hippocampus	0	n.s.	n.s.	7.79	−2.72×10^−8^	−1.59×10^−8^ to −3.98×10^−8^
		Thalamus	0	n.s.	n.s.	0	n.s.	n.s.

Significant regions are reported as a percentage of vertices affected (%), average effect size (Cohen’s d) and range of effect sizes of differences in Jacobian Determinant (surface dilation ratio) and radial distance (thickness).

n.s., not significant.

In our analysis of radial distance, which reflects thickness, former American football players exhibited significant negative values bilaterally in the amygdala, indicating reduced thickness, compared to unexposed asymptomatic controls. Specifically, in the amygdala, left-side reductions in thickness were observed in the basolateral, basomedial and lateral nuclei (Cohen’s d = −0.419, *P* = 0.001), while right-side reductions were found in the basolateral, basomedial, centromedial and lateral nuclei (Cohen’s d = −0.269, *P* = 0.01). No other regions reached statistical significance; see Fig. 1 and Table 2 for additional details.

### Association with age

**Figure 2:**
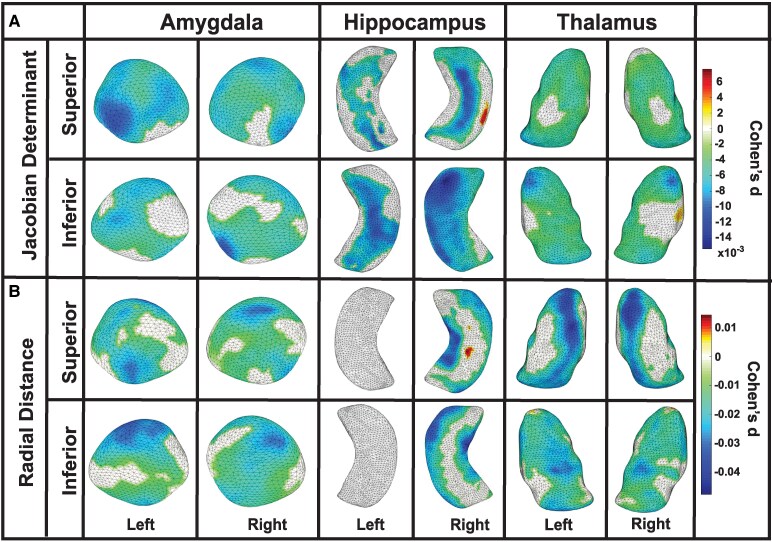
**Age-related structural decline in the amygdala, hippocampus and thalamus among former football players**. Partial correlation analyses demonstrating interaction with age in former American football players (*N* = 163). (**A**) Jacobian determinant values revealed significant age-related surface area contractions in the amygdala, hippocampus and thalamus. In the amygdala, left-side contractions were observed in the basolateral, basomedial, centromedial and lateral nuclei (Cohen’s d = −0.007, *P* = 0.04), with similar right-side contractions (Cohen’s d = −0.006, *P* = 0.04). In the hippocampus, left-side contractions were noted in the CA1, CA2, CA3/DG and subiculum subregions (Cohen’s d = −0.008, *P* = 0.02), and right-side contractions were observed in the same subregions (Cohen’s d = −0.007, *P* = 0.03). In the thalamus, left-side contractions were found in the anteroventral, ventral, medial-lateral and pulvinar subregions (Cohen’s d = −0.004, *P* = 0.04), while right-side contractions were restricted to the anteroventral, ventral, medial-lateral and pulvinar subregions (Cohen’s d = −0.004, *P* = 0.04). (**B**) In the analysis of radial distance in the amygdala, left-sided reductions were observed in the basolateral, basomedial, centromedial and lateral nuclei (Cohen’s d = −0.019, *P* = 0.03), and right-sided reductions were seen in the same nuclei (Cohen’s d = −0.016, *P* = 0.04). In the hippocampus, right-sided reductions were noted in the CA1, CA2, CA3/DG and subiculum subregions (Cohen’s d = −0.022, *P* = 0.03). In the thalamus, left-sided reductions were observed in the anteroventral, ventral, medial, lateral, and pulvinar subregions (Cohen’s d = −0.021, *P* = 0.04), and right-sided reductions were seen in the same subregions (Cohen’s d = −0.018, *P* = 0.03). DG, dentate gyrus.

**Figure 3:**
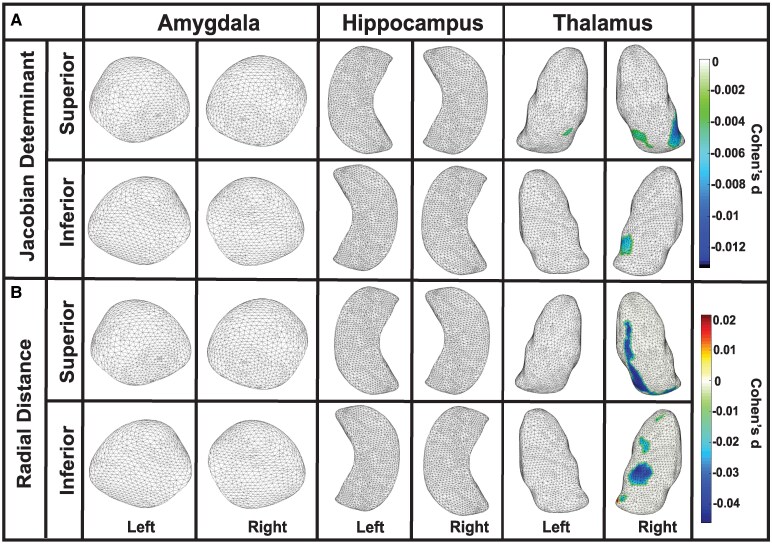
**Age-related thalamic changes in unexposed controls**. Partial correlation analyses demonstrating interaction with age in unexposed asymptomatic controls (*N* = 53). (**A**) Jacobian determinant values revealed significant interactions with age in the thalamus bilaterally, indicating surface area contractions, specifically in the ventral and pulvinar subregions (left: Cohen’s d = −0.006, *P* < 0.0001; right: Cohen’s d = −0.008, *P* < 0.01). (**B**) An interaction with age was also observed for radial distance values in the right thalamus, specifically in the ventral, medial, lateral and pulvinar subregions (Cohen’s d = −0.031, *P* < 0.01), indicating an association between older age and reduced thickness.

An interaction with age was observed among former American football players for Jacobian determinant values bilaterally in the amygdala, hippocampus and thalamus, indicating a negative association between older age and surface area contraction in these regions. In the amygdala, left-side contractions were seen in the basolateral, basomedial, centromedial and lateral nuclei (Cohen’s d = −0.007, *P* = 0.04), and right-side contractions were found in the basolateral, basomedial, centromedial and lateral nuclei (Cohen’s d = −0.006, *P* = 0.04). In the hippocampus, left-side contractions were observed in the CA1, CA2, CA3/DG and subiculum subregions (Cohen’s d = −0.008, *P* = 0.02), and right-side contractions were noted in the CA1, CA2, CA3/DG and subiculum subregions (Cohen’s d = −0.007, *P* = 0.03). In the thalamus, left-side contractions were observed in the anteroventral, ventral, medial-lateral and pulvinar subregions (Cohen’s d = −0.004, *P* = 0.04), and right-side contractions were noted in anteroventral, ventral, medial-lateral and pulvinar subregions (Cohen’s d = −0.004, *P* = 0.04); see Fig. 2 and Table 2 for further details. Among the unexposed asymptomatic control group, a significant interaction with age was observed for Jacobian determinant values bilaterally in the thalamus, indicating a negative association between older age and surface area contractions. Contractions were observed in the left (Cohen’s d = −0.006, *P* < 0.0001) and right (Cohen’s d = −0.008, *P* < 0.01) thalamus, specifically in the ventral and pulvinar subregions. No other regions showed significant interactions. For further details, see Fig. 3 and Table 2.

An interaction with age was observed among former American football players for radial distance values in the amygdala bilaterally, right hippocampus and thalamus bilaterally, indicating a negative association between older age and reduced thickness in these regions. In the amygdala, left-side reductions were observed in the basolateral, basomedial, centromedial and lateral nuclei (Cohen’s d = −0.019, *P* = 0.03), while right-side reductions were found in the basolateral, basomedial, centromedial and lateral nuclei (Cohen’s d = −0.016, *P* = 0.04). In the hippocampus, right-side reductions were observed in the CA1, CA2, CA3/DG and subiculum subregions (Cohen’s d = −0.022, *P* = 0.03). In the thalamus, left-side reductions were found in the anteroventral, ventral, medial, lateral and pulvinar subregions (Cohen’s d = −0.021, *P* = 0.04), while right-side reductions were observed in the anteroventral, ventral, medial, lateral and pulvinar subregions (Cohen’s d = −0.018, *P* = 0.03). No other regions showed significant interactions; see Fig. 2 and Table 2 for details. In unexposed asymptomatic controls, a negative interaction with age was observed for radial distance values in the right thalamus, specifically in the ventral, medial, lateral and pulvinar subregions (Cohen’s d = −0.031, *P* < 0.01), indicating an association between older age and reduced thickness. No other regions showed significant interactions; see Fig. 3 and Table 2 for details.

### Associations with exposure factors

#### Age of first exposure to football

Among former American football players, we observed an interaction between the age of first exposure to tackle football and positive Jacobian determinant values in the left hippocampus and bilaterally in the thalamus, reflecting an association between an older age of first exposure to tackle football and surface area expansion in these regions. Specifically, in the left hippocampus, surface area expansion was seen in the CA2 subregion (Cohen’s d = 0.026, *P* < 0.001). In the thalamus, surface area expansion was observed in the left anteroventral, ventral and medial subregions (Cohen’s d = 0.009, *P* < 0.01) and the right ventral subregion (Cohen’s d = 0.010, *P* < 0.001). Interpreted inversely, these findings indicate that an earlier age of first exposure to tackle football is associated with surface area contractions in these regions. In short, these findings indicate worse outcomes the earlier one participates in American tackle football and therefore earlier exposure to RHI. No other regions showed significant interactions.

**Figure 4:**
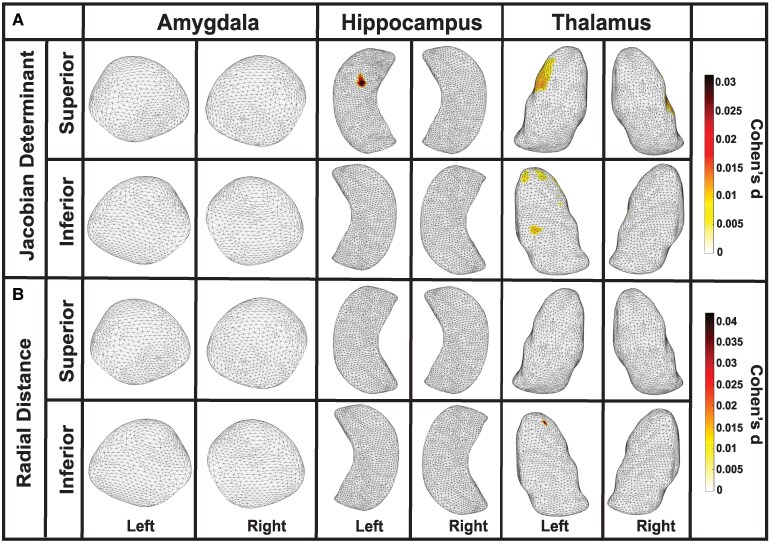
**Age of first football exposure relates to hippocampal and thalamic morphology**. Partial correlation analyses demonstrating interaction with the age of first exposure to tackle football (*N* = 163). (**A**) Jacobian determinant values revealed significant interactions with the age of first exposure to tackle football in the left hippocampus, showing surface area expansion in the CA2 subregion (Cohen’s d = 0.026, *P* < 0.001). In the thalamus, surface area expansion was observed in the left anteroventral, ventral, and medial subregions (Cohen’s d = 0.009, *P* < 0.01), as well as the right ventral subregion (Cohen’s d = 0.010, *P* < 0.001). (**B**) An interaction with age of first exposure was also observed for radial distance values, with increased thickness noted in the ventral subregion of the left thalamus (Cohen’s d = 0.04, *P* < 0.0001). For clarity, positive coefficients reflect expansion with higher age at first exposure; the inverse holds for earlier exposure (contraction).

In the analysis of radial distance, an interaction with the age of first exposure to tackle football was observed in the left thalamus, where increased thickness was noted in the ventral subregion (Cohen’s d = 0.04, *P* < 0.0001) to be associated with an older age of first exposure; see Fig. 4 and Table 2. In short, a younger age of first exposure to tackle football corresponds to decreased thickness in the same regions. Again, this indicates worse outcomes with a younger age of exposure to tackle football.

#### CHII-frequency, linear acceleration and rotational force

**Figure 5:**
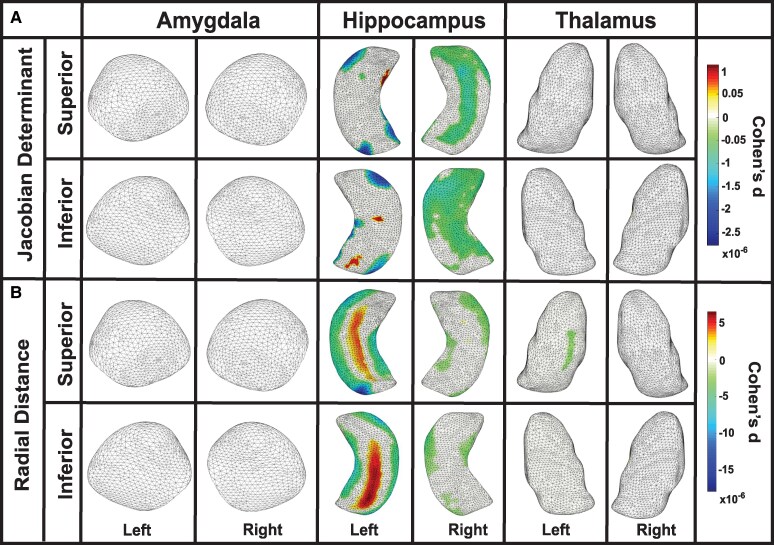
**Cumulative linear acceleration burden is linked to hippocampal and thalamic morphometric loss**. Partial correlation analyses demonstrating interaction with CHII linear acceleration (*N* = 163). (**A**) Jacobian determinant values showed significant interactions with CHII linear acceleration in the hippocampus, indicating surface area contraction. Left-side contractions were observed in the CA1, CA2, CA3/DG and subiculum subregions (Cohen’s d = −1.22E-06, *P* < 0.01), while right-side contractions were found in the same subregions (Cohen’s d = −5.61E-07, *P* = 0.03). (**B**) Radial distance analysis revealed an interaction with linear acceleration, showing reductions in thickness bilaterally in the hippocampus (CA1, CA2, CA3/DG, and subiculum subregions (left: Cohen’s d = −2.49E-06, *P* = 0.03; right: Cohen’s d = −2.19E-06, *P* = 0.01), as well as in the left thalamus, specifically in the medial and pulvinar subregions (Cohen’s d = −2.18E-06, *P* < 0.001). CHII, cumulative head impact index; DG, dentate gyrus.

Among former American football players, no significant interactions were observed between Jacobian determinant or radial distance values and CHII frequency in our regions of interest. However, we identified a bilateral interaction between CHII linear acceleration and negative Jacobian determinant values in the hippocampus, suggesting an association between increased linear acceleration and surface area contraction. Specifically, in the hippocampus, left-side contractions were observed in the CA1, CA2, CA3/DG and subiculum subregions (Cohen’s d = −1.22E-06, *P* < 0.01), while right-side contractions were found in the CA1, CA2, CA3/DG and subiculum subregions (Cohen’s d = −5.61E-07, *P* = 0.03); see Fig. 5 and Table 2.

In the analysis of radial distance, an interaction with linear acceleration was observed bilaterally in the hippocampus, with left-side reductions in thickness in the CA1, CA2, CA3/DG and subiculum subregions (Cohen’s d = −2.49E−06, *P* = 0.03) and right-side reductions in the CA1, CA2, CA3/DG and subiculum subregions (Cohen’s d = −2.19E−06, *P* = 0.01). Additionally, a reduction in thickness was observed in the left thalamus, specifically in the medial and pulvinar subregions (Cohen’s d = −2.18E−06, *P* < 0.001); see Fig. 5 and Table 2.

**Figure 6:**
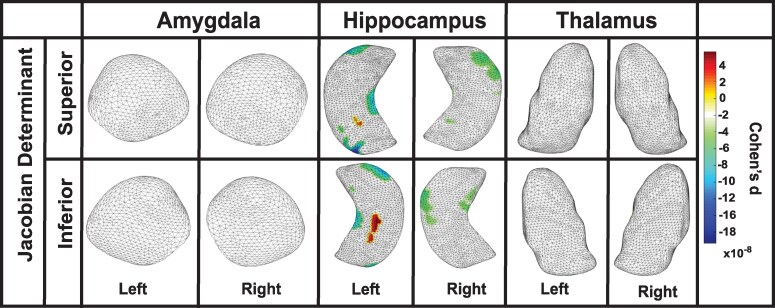
**Cumulative rotational force burden predicts hippocampal thinning**. Partial correlation analyses demonstrating interaction with CHII rotational force (*N* = 163). Analysis of radial distance revealed an interaction with CHII rotational force, showing an association between increased rotational force and reduced cortical thickness in the hippocampus. Specifically, left-side reductions in thickness were observed in the CA1, CA2, CA3/DG, and subiculum subregions (Cohen’s d = −4.65E−08, *P* < 0.01), while right-side reductions were noted in the CA1, CA2 and subiculum subregions (Cohen’s d = −2.72E−08, *P* < 0.01). CHII, cumulative head impact index; DG, dentate gyrus.

No significant interactions were observed between Jacobian determinant values and CHII rotational force. However, a bilateral interaction between rotational force and negative radial distance values was found in the hippocampus, indicating an association between increased rotational force and reduced thickness. Specifically, left-side reductions in thickness were observed in the CA1, CA2, CA3/DG and subiculum subregions (Cohen’s d = −4.65E-08, *P* < 0.01), while right-side reductions were observed in the CA1, CA2 and subiculum subregions (Cohen’s d = −2.72E-08, *P* < 0.01); see Fig. 6 and Table 2. No other regions showed significant interactions.

#### Group-level differences with TES

No significant differences were observed in either Jacobian determinant or radial distance values between former American football players with or without a TES diagnosis within our preselected regions of interest.

## Discussion

This study provides new evidence of structural brain alterations in former American football players with a history of RHI. Using shape analysis, we identified significant contractions in the surface area of the amygdala and hippocampus, as well as reduced amygdalar thickness, findings that align with post-mortem reports of CTE pathology. These morphological changes were further supported by key exposure factors, including the age of first exposure to tackle football and cumulative head impact estimates. Importantly, these structural findings are consistent with prior longitudinal and epidemiological studies demonstrating that greater RHI exposure is associated with worse long-term clinical outcomes, including cognitive impairment, neurobehavioural dysregulation and increased risk of dementia.^23,49,68-73^ Thus, while the present study is cross-sectional and cannot directly assess clinical decline, the observed morphometric alterations align with the broader literature, suggesting that early and prolonged exposure to RHI contributes to later-life neurodegenerative risk.^18,23^ Additionally, age-related surface area contractions and structural thinning were more pronounced in former American football players compared to unexposed asymptomatic controls, suggesting potential interactions between aging and RHI-related neurodegeneration. Although we did not find significant differences based on TES diagnosis, this may reflect the heterogeneity and limited sensitivity of current diagnostic criteria rather than the absence of underlying structural changes. Overall, these findings underscore the importance of continued research using advanced imaging techniques to refine *in vivo* biomarkers for CTE and related disorders.

### Morphometry in former American football players

Our use of surface-based shape analysis offers insights beyond traditional volumetry. Whereas volumetry captures global atrophy, shape analysis pinpoints changes in specific subregions, providing greater anatomical precision and clearer links to clinical, behavioural and disease-related outcomes. Beginning with our group-level analysis, we found that former American football players exhibited surface area contraction bilaterally in the amygdala compared to unexposed asymptomatic controls. Specifically, these contractions were localized to the basolateral and basomedial regions. Notably, these regions of the amygdala play critical roles in regulating anxiety, reward behaviour and fear response behaviours, respectively.^74^ Dysfunction of the amygdala has been linked to impulsivity,^75^ depression^76,77^ and suicidality,^71,78,79^ which are common behavioural patterns observed in former American football players.^28,80^ In the hippocampus, we found that former American football players exhibited bilateral surface area contractions localized in the fissure, CA1, CA3 and part of the tail. The ventral aspect of these regions, in particular, is known to play a role in expressing affective and motivated behaviours.^74^ Hippocampal deficits have additionally been implicated in depression and suicidality,^71^ again providing insight into the potential link between brain and behavioural changes commonly observed in former American football players.^71^ Notably, we did not find significant group-level differences in thalamic surface area dilation. This result was somewhat unexpected, given that previous studies have reported reductions in gross thalamic volume, particularly in retired fighters who have experienced RHI.^81^ The absence of such findings here may reflect differences in analytic sensitivity, with volumetry detecting diffuse global atrophy while shape analysis highlights focal changes, or sport-specific differences in thalamic vulnerability between fighters and football players. For example, fighters are subject to frequent high-magnitude blows to the head, including rotational forces, that may render the thalamus more vulnerable to degeneration than in football players, whose exposures are typically more repetitive but of somewhat lower magnitude. Future research with larger samples and complementary imaging modalities may be necessary to clarify the role of thalamic degeneration in this group and determine whether other structural changes may be more predictive of neurodegenerative processes in athletes with a history of RHI.

Regarding the analysis of radial distance, we found that former American football players exhibited reduced thickness of the amygdala bilaterally compared to unexposed asymptomatic controls. Thus, in former American football players, we were able to detect surface area contraction bilaterally in the amygdala and hippocampus, and reduced thickness bilaterally in the amygdala. These results not only replicate our previous volumetric findings of the amygdala and hippocampus showing gross volumetric reductions but also allow us to pinpoint where these specific morphological changes are occurring on the surfaces of these regions, which notably aligns with the neuropathology of CTE at post-mortem.^9,12-14,19-22,41,42^ By comparing both surface area dilation and radial distance, we can better understand whether changes in surface area, thickness, or a combination of both primarily drive the previously observed volumetric differences.^32,82^ In this analysis, we observed a mixed pattern of results, indicating that the structural alterations may not be limited to one specific measure. This nuanced approach allows us to differentiate between various morphological changes, providing a more comprehensive understanding of the underlying structural dynamics in former athletes with RHI exposure.

### Associations between morphology and age

Among former American football players, an interaction between age and surface dilation was observed. Increased age was associated with surface area contraction bilaterally in the amygdala, hippocampus and thalamus. Among unexposed asymptomatic controls, an interaction between increased age and surface area contraction was only observed bilaterally in the thalamus.

In former American football players, increased age was linked to reduced thickness in the bilateral amygdala, right hippocampus and bilateral thalamus. In contrast, unexposed asymptomatic controls exhibited age-related thinning solely in the right thalamus. These findings highlight the interaction between natural aging and potential neurodegenerative processes. Given the increased likelihood of former American football players being diagnosed with CTE at post-mortem, it is plausible that these changes reflect neurodegeneration associated with tau protein aggregation. Unlike healthy aging, conditions such as CTE and Alzheimer’s disease are marked by accelerated brain atrophy driven by mechanisms such as neuroinflammation and the accumulation of neurotoxic proteins, including tau tangles in CTE and amyloid beta plaques in Alzheimer’s.^11,12,14,19,42^ This acceleration leads to exacerbated gray matter loss and cortical thinning, which we have previously reported in this cohort.^29^ However, our previous analyses did not identify an age-by-group interaction between former American football players and unexposed asymptomatic controls when examining gross hippocampal and amygdalar volumes. In contrast, this more refined analysis, which captures subtle changes at smaller vertices, successfully detected significant age-related associations in both groups.

### Associations with exposure factors

The first RHI-related exposure factor of interest was the age of first exposure to tackle football. It has previously been shown that the age of first exposure to tackle football, particularly before age 12, is linked to a higher likelihood of depression, behavioural regulation, executive function and apathy,^51^ as well as decreased cortical thickness later in life.^52^ Although self-reported cumulative measures of RHI burden (e.g. total years of play) were available, we analyzed age at first exposure as an independent predictor because it uniquely indexes developmental timing/early-life susceptibility and directly tests whether earlier exposure relates to subregional brain morphology. Moreover, total years of play are already incorporated into the CHII; modelling it separately would be redundant and could introduce collinearity without adding interpretive value. Our analysis of surface area dilation among former American football players found no significant associations in the amygdala. However, we did find slight associations in the left hippocampus and bilaterally in the thalamus. In those areas, an older age of first exposure was associated with surface area expansion. This indicates that a younger age of first exposure leads to surface area contraction in those areas. Specifically, those changes are seen in the CA1 region of the left hippocampus and the lateral nuclei of the thalamus bilaterally. In our analysis of thickness among former American football players, we found an association only between an older age of first exposure and increased thickness in the left thalamus. Thus, within the former American football player group, we demonstrated that an older age of first exposure to tackle football was associated with surface area expansion in the left hippocampus and bilateral thalamus, as well as increased thickness of the left thalamus. By extension, a younger age of first exposure was associated with surface area contraction and reduced thickness in those areas. These findings indicate a protective effect of starting to play American football later in life, particularly in the thalamus, or might suggest that having larger structures confers resilience to play-related alterations. Previous research has demonstrated that a younger age of first exposure to RHI in tackle football was linked to decreased right thalamic volume in former professional American football players, which supports our findings.^16^

In estimates of career head impacts, we did not find any associations between CHII scores of frequency and surface area dilation or thickness in any of our regions of interest. However, we found an association with surface area contraction bilaterally in the hippocampus for CHII scores of linear acceleration. This indicates that greater scores of linear acceleration were associated with surface area contraction, specifically in the tail, CA1 and CA3 regions. We also found an association between CHII scores of linear acceleration and decreased thickness bilaterally in the hippocampus and the left thalamus. Thus, former American football players with higher estimate scores of linear acceleration demonstrated surface area contraction in the hippocampus bilaterally and reduced thickness of the hippocampus bilaterally and left thalamus. We did not find associations with surface area dilation across our regions of interest for CHII scores of rotational force. However, we did observe an association with reduced thickness in the hippocampus. Thus, former American football players with higher estimate scores of rotational forces demonstrated decreased thickness of the hippocampus bilaterally. Together, these findings link higher CHII scores of linear acceleration and rotational force to abnormalities in the morphology of the hippocampus and thalamus. This adds to previous research demonstrating a crucial role of cumulative head impact intensity experienced during American football and brain regions associated with the neuropathology of CTE pathogenesis.^83,84^ It is important to note that our previous work did not find an interaction between CHII measures and gross hippocampal and amygdala volumes.^29^ This earlier analysis focused on broader structural measures, which may have overlooked subtle variations. In contrast, the current approach is more anatomically specific, allowing for a detailed examination of these regions at the level of smaller vertices. By assessing local surface changes, we can better capture finer structural alterations that may be missed when analyzing these regions in their entirety. This methodological advancement enhances our ability to detect subtle changes that may be relevant to the effects of RHI.

### Lack of associations with TES diagnosis

Lastly, we sought to investigate whether we could identify group-level differences in structural morphology between former American football players with and without a TES diagnosis. Notably, we found no significant differences between these groups across our regions of interest in Jacobian determinant and radial distance analyses. This null finding is particularly surprising given that the amygdala and hippocampus are critical regions involved in the clinical symptomatology of TES, including cognitive impairment and neurobehavioural dysregulation.^28,85^ While this may suggest a lack of group-level morphological differences, it is also important to acknowledge that TES itself may not fully capture the full range of neurobiological alterations associated with RHI. The diagnostic criteria for TES could potentially be limited in its ability to reflect these changes, as it may not encompass all the subtle neuroanatomical shifts that are present in individuals with head trauma but without a formal TES diagnosis. Furthermore, the heterogeneity in the manifestation of TES symptoms, including the timing and severity of cognitive and neurobehavioural impairments, might contribute to variability in structural imaging findings, making it challenging to detect consistent group-level differences. Alternatively, the absence of TES-related differences may reflect the nonspecific nature of both constructs: TES represents a broad and heterogeneous clinical syndrome, while shape metrics capture focal structural alterations. A direct correspondence between the two, therefore, may not be expected.

### Limitations

The generalizability of our findings is limited by certain factors. Notably, our study sample included only male former American football participants, which restricts our ability to extend the observed associations of RHI exposure to other sports, female athletes, gender-diverse athletes or even current athletes. Second, our sample of former American football players spans a wide range of career years (1952–2007), during which the sport has undergone significant changes. We acknowledge that gameplay has evolved over this time, particularly with rule modifications related to the intensity of play (e.g. helmet-to-helmet contact, kickoff rules and protections for quarterbacks and defenseless receivers) and increased safety measures, such as enhanced concussion protocols and head impact guidelines. Third, concerning cumulative RHI exposure, it is essential to note that CHII scores are estimates derived from helmet accelerometer data from the high school and collegiate levels, as well as the self-reported position and number of seasons played. This is because there is no available helmet accelerometer data, to our knowledge, in professional American football. Fourth, given the *in vivo* focus of this study, we do not have post-mortem data for our participants that could determine CTE pathology and diagnosis.

## Conclusions

Our findings provide new insights into the structural brain alterations associated with RHI in former American football players. We observed significant surface area contractions and reduced thickness in the amygdala and hippocampus, aligning with regions implicated in the neuropathology of CTE. These changes were further influenced by age and exposure factors, including the age of first exposure to tackle football and cumulative head impact estimates, suggesting a potential role in neurodegenerative risk. Importantly, our results highlight the utility of surface-based morphometry in detecting subtle structural changes that may not be captured by traditional volumetric analyses. However, the lack of significant group differences based on TES diagnosis underscores the complexity of linking clinical symptoms to underlying neurodegenerative pathology. Future studies with larger cohorts and multimodal imaging approaches are necessary to further refine *in vivo* biomarkers for CTE and improve early detection and intervention strategies for at-risk athletes.

## Data Availability

Data from the DIAGNOSE CTE Research Project is accessible to qualified researchers via the Federal Interagency Traumatic Brain Injury Research (FITBIR) Informatics System, managed by the NIH Center for Information Technology: https://fitbir.nih.gov/content/access-data. In addition, project-specific data, including those reported in this study, will be available through a dedicated data-sharing portal. Researchers interested in accessing the data should contact Dr. Hector Arciniega at Hector.Arciniega@nyulangone.org.

## Appendix

### DIAGNOSE CTE Research Project Team

Eric Reiman, M.D.; Yi Su, Ph.D.; Kewei Chen, Ph.D.; Hillary Protas, Ph.D.; Connie Boker, M.B.A.; Michael L. Alosco, Ph.D.; Rhoda Au, Ph.D.; Robert C. Cantu, Ph.D.; Lindsay Farrer, Ph.D.; Robert Helm, M.D.; Douglas I. Katz, M.D.; Neil Kowall, M.D.; Jesse Mez, M.D.; Gustavo Mercier, M.D., Ph.D.; James Otis, M.D.; Robert A. Stern, Ph.D.; Jason Weller, M.D.; Irene Simkin, M.S.; Alondra Andino, B.A.; Shannon Conneely, B.A.; Courtney Diamond, M.B.A.; Tessa Fagle, B.A.; Olivia Haller, B.A.; Tennyson Hunt, M.B.A.; Nicole Gullotti, M.B.A.; Megan Mariani, B.S., B.A.; Brian Mayville, B.S.; Kathleen McLaughlin, B.A.; Mary Nanna, B.A.; Taylor Platt, M.P.H.; Surya Pulukuri, B.A.; Fiona Rice, M.P.H.; Madison Sestak, B.S.; Michael McClean, Sc.D.; Yorghos Tripodis, Ph.D.; Douglas Annis, M.S.; Christine Chaisson, M.P.H.; Diane B. Dixon; Carolyn Finney, B.A.; Kerrin Gallagher, M.P.H.; Kaitlin Hartlage, M.P.H.; Jun Lu, M.S.; Brett Martin, M.S.; Emmanuel Ojo, M.P.H.; Joseph N. Palmisano, M.A., M.P.H.; Brittany Pine, B.A., B.S.; Janani Ramachandran, M.S.; Sylvain Bouix, Ph.D.; Jennifer Fitzsimmons, M.D.; Alexander P. Lin, Ph.D.; Inga K. Koerte, M.D., Ph.D.; Ofer Pasternak, Ph.D.; Martha E. Shenton, Ph.D.; Hector Arcinieago, Ph.D.; Tashrif Billah, M.S.; Elena Bonke, M.S.; Katherine Breedlove, Ph.D.; Eduardo Coello, Ph.D.; Michael J. Coleman, M.A.; Leonhard Jung; Huijun Liao, B.S.; Maria Loy, M.B.A., M.P.H.; Elizabeth Rizzoni, B.A.; Vivian Schultz, M.D.; Annelise Silva, B.S.; Brynn Vessey, B.S.; Tim L.T. Wiegand; Sarah Banks, Ph.D.; Charles Bernick, M.D.; Jason Miller, Ph.D.; Aaron Ritter, M.D.; Marwan Sabbagh, M.D.; Raelynn de la Cruz; Jan Durant; Morgan Golceker; Nicolette Harmon; Kaeson Kaylegian; Rachelle Long; Christin Nance; Priscilla Sandoval; Robert W. Turner, Ph.D.; Kenneth L. Marek, M.D.; Andrew Serrano, M.B.A.; Charles H. Adler, M.D., Ph.D.; David W. Dodick, M.D.; Yonas Geda, M.D., MSc; Jennifer V. Wethe, Ph.D.; Bryce Falk, R.N.; Amy Duffy; Marci Howard; Michelle Montague; Thomas Osgood; Debra Babcock, M.D., Ph.D.; Patrick Bellgowan, Ph.D.; Laura Balcer, M.D., M.S.C.E.; William Barr, PhD.; Judith Goldberg, Sc.D.; Thomas Wisniewski, M.D.; Ivan Kirov, Ph.D.; Yvonne Lui, M.D.; Charles Marmar, M.D.; Lisena Hasanaj; Liliana Serrano; Alhassan Al-Kharafi; Allan George; Sammie Martin; Edward Riley; William Runge; Jeffrey L. Cummings, M.D., ScD; Elaine R. Peskind, M.D.; Elizabeth Colasurdo; Daniel S. Marcus, Ph.D.; Jenny Gurney, M.S.; Richard Greenwald, Ph.D.; Keith A. Johnson, M.D.
